# *BDNF* coexpresses with *MTOR* and is associated with muscle fiber size, lean mass and power-related traits

**DOI:** 10.1007/s00421-025-05804-3

**Published:** 2025-04-29

**Authors:** Celal Bulgay, Erdal Zorba, Hasan H. Kazan, Işık Bayraktar, Merve Uca, Mehmet A. Ergün, George John, Rinat A. Yusupov, Rinat I. Sultanov, Ekaterina A. Semenova, Andrey K. Larin, Nikolay A. Kulemin, Edward V. Generozov, Ildus I. Ahmetov

**Affiliations:** 1 Sports Science Faculty, Bingol University, Bingol, Türkiye; 2 Sport Sciences Faculty, Gazi University, Ankara, Türkiye; 3 Department of Medical Biology, Gulhane Faculty of Medicine, University of Health Sciences, Ankara, Türkiye; 4 Sports Science Faculty, Alanya Alaaddin Keykubat University, Alanya, Türkiye; 5 School of Physical Education and Sports, İstanbul Esenyurt University, Istanbul, Türkiye; 6 Department of Medical Genetics, Faculty of Medicine, Gazi University, Ankara, Türkiye; 7 Transform Specialist Medical Centre, Dubai, United Arab Emirates; 8 Department of Physical Culture and Sport, Kazan National Research Technical University Named After A.N. Tupolev-KAI, Kazan, Russia; 9 Department of Molecular Biology and Genetics, Lopukhin Federal Research and Clinical Center of Physical-Chemical Medicine of Federal Medical Biological Agency, Moscow, Russia; 10 Research Institute of Physical Culture and Sport, Volga Region State University of Physical Culture, Sport and Tourism, Kazan, Russia; 11 Sports Genetics Laboratory, St Petersburg Research Institute of Physical Culture, St Petersburg, Russia; 12 Laboratory of Genetics of Aging and Longevity, Kazan State Medical University, Kazan, Russia; 13 Research Institute for Sport and Exercise Sciences, Liverpool John Moores University, Liverpool, UK

**Keywords:** DNA, Polymorphism, SNP, Gene expression, Sprint, Sports

## Abstract

**Purpose:**

Recent research suggests a link between brain-derived neurotrophic factor (BDNF) and the mTOR signaling pathway, a key regulator of protein synthesis and muscle growth. However, it remains unclear whether BDNF influences muscle growth and power performance. Our study aimed to investigate the relationship between the expression of *BDNF* and *MTOR* genes in human skeletal muscle and examine the association between genetically predicted higher expression of the *BDNF* gene and muscle fiber size, lean mass, power performance, and power athlete status.

**Methods:**

The study involved 456,382 subjects, including 285 athletes, 112 physically active individuals with muscle fiber composition data, 291 sedentary individuals with gene expression data, 5451 controls, and 450,243 UK Biobank participants. The muscle fiber composition was evaluated using immunohistochemistry, while gene expression analysis was performed using RNA sequencing. *BDNF* genotyping was carried out using real-time PCR or microarrays.

**Results:**

We found that *BDNF* gene expression was positively associated with *MTOR* gene expression in the vastus lateralis (*p* < 0.0001). Furthermore, genetically predicted higher *BDNF* expression (i.e., carriage of the C allele of the rs6265 (Val66Met) *BDNF* polymorphism) was positively associated with the cross-sectional area of fast-twitch muscle fibers in athletes (*p* = 0.0069), appendicular lean mass (*p* = 2.6 × 10⁻⁷), personal best scores of power athletes (*p* = 0.029), and power athlete status (*p* = 0.0056).

**Conclusion:**

Our study demonstrates a positive correlation between *BDNF* and *MTOR* gene expression in human skeletal muscle, with genetically predicted higher *BDNF* expression associated with greater muscle fiber size, lean mass, enhanced power performance, and power athlete status.

**Supplementary Information:**

The online version contains supplementary material available at 10.1007/s00421-025-05804-3.

## Introduction

Skeletal muscle hypertrophy is a critical physiological adaptation that enhances physical performance. An increase in muscle size is directly associated with improvements in maximal strength, sprinting, and jumping performance (Seynnes et al. 2007; Handsfield et al. 2017; Bchini et al. 2023). Promoting muscle hypertrophy through interventions such as resistance training is also essential for mitigating the effects of sarcopenia and preserving physical function in older adults (Peterson et al. 2011).

At the tissue level, skeletal muscle hypertrophy is characterized by increased muscle fiber size, primarily driven by increased satellite cell abundance and myonuclear accretion (Roberts et al. 2023). At the molecular level, hypertrophy is regulated by a complex network of signaling pathways and gene expression changes (Glass 2003; Kollias and McDermott 2008; Roberts et al. 2023; Baumert et al. 2024). Among these, the mechanistic target of rapamycin kinase (mTOR) pathway is a central regulator of protein synthesis and ribosomal biogenesis in response to resistance exercise and nutrient availability (Schiaffino et al. 2021).

Both environmental (i.e., resistance training, nutrition, microbiome, etc.) and heritable factors play crucial roles in determining muscle fiber size. The animal studies suggest that heritable factors contribute to 19–32% of the variation in muscle fiber size (Rivero et al. 2001; Liu et al. 2021), while human studies report heritability estimates of 52% for lean mass (Arden and Spector 1997) and 55–81% for skeletal muscle mass (Livshits et al. 2016; You et al. 2018). Heritable factors may encompass DNA sequence variations (i.e., genetic variants), including single-nucleotide polymorphisms (SNPs), insertions and deletions (indels), and structural variations, which can influence gene expression and protein structure and, as a consequence, exercise-related traits (Boulygina et al. 2020). Recently, efforts have been made to identify genetic variants associated with muscle fiber cross-sectional area (Guilherme et al. 2024; Çığırtaş et al. 2024) and related phenotypes, such as appendicular lean mass (Pei et al. 2020), sarcopenia (Semenova et al. 2023), strength (Gabbasov et al. 2013; Tikkanen et al. 2018; Moreland et al. 2022; Kikuchi et al. 2022), power athlete status (Maciejewska-Skrendo et al. 2019) and testosterone levels (Ahmetov et al. 2014; Ruth et al. 2020; Guilherme et al. 2022a).

Among the factors of interest related to skeletal muscle hypertrophy, brain-derived neurotrophic factor (BDNF) has garnered attention for its connection to the mTOR signaling pathway (Chen et al. 2009; Suijo et al. 2013; Hang et al. 2017; Giacco et al. 2022) and its association with power, strength, and sprint performance (Murtagh et al. 2020; Guilherme et al. 2022b). BDNF, a key neurotrophic factor widely expressed in the brain and central nervous system, promotes neuronal survival, growth, and plasticity through the activation of its receptors, tropomyosin receptor kinase B (TrkB) and p75 neurotrophin receptor (Li et al. 2022). The reduced BDNF levels are associated with increased risks of neurodegenerative, psychiatric, and cardiovascular diseases (Pisani et al. 2023; Lei et al. 2024). The role of BDNF in enhancing synaptic transmission and promoting long-term potentiation underscores its significance in motor learning and neuromuscular performance (De Vincenti et al. 2019; Miranda et al. 2019; Andreska et al. 2020). Importantly, exercise enhances cognitive function, potentially through mechanisms such as the elevated expression of BDNF in the brain (Rasmussen et al. 2009; Wrann et al. 2013), although this represents just one of several possible pathways.

BDNF is also expressed in skeletal muscle, where its gene expression is influenced by exercise and metabolic conditions (Matthews et al. 2009; Rentería et al. 2022). In skeletal muscle, BDNF contributes to muscle repair, adaptation, and energy homeostasis by regulating processes like mitochondrial biogenesis and fatty acid oxidation through the activation of AMP-activated protein kinase (AMPK) signaling pathways (Mousavi et al. 2004; Matthews et al. 2009; Pedersen and Febbraio 2012; Rentería et al. 2022). It also promotes muscle regeneration by enhancing satellite cell proliferation and differentiation (Clow and Jasmin 2010). The absence of muscle-specific *Bdnf* gene reduces the motor endplate size in the extensor digitorum longus muscle. It induces a shift from fast to slow phenotypes in fast IIb (glycolytic) muscle fibers in mice. Conversely, overexpression of *Bdnf* in adult mouse muscles enhances the expression of fast-type genes and increases the proportion of fast IIb muscle fibers (Delezie et al. 2019). A recent meta-analysis found that lower BDNF levels were strongly associated with decreased muscle protein synthesis, increased protein breakdown, mitochondrial dysfunction, heightened oxidative stress, and impaired neuromuscular junction integrity (Susanto et al. 2024).

Genetic variation is considered one of the factors influencing *BDNF* gene expression. Specifically, the common C (Val) allele of the rs6265 (Val66Met) polymorphism in the *BDNF* gene has been linked to increased *BDNF* expression in both skeletal muscle (de Assis et al. 2021) and heart muscle (GTEx portal, 2025). Conversely, the minor T (Met) allele has been associated with decreased *BDNF* expression, which is consequently linked to reduced sprint and power performance (Murtagh et al. 2020), poorer episodic memory (Egan et al. 2003), and a higher risk of major depressive disorder (Wang et al. 2023). These findings suggest that the *BDNF* rs6265 polymorphism may predict *BDNF* gene expression and cognitive- and exercise-related phenotypes.

These findings suggest that BDNF could influence skeletal muscle hypertrophy and power performance through its association with the mTOR signaling pathway and its impact on muscle repair. However, further investigation is needed to fully understand its role in this process and confirm its potential as a modulator of muscle growth. Therefore, our study aimed to investigate the relationship between the expression of *BDNF* and *MTOR* genes in human skeletal muscle and to examine the association between genetically predicted higher expression of the *BDNF* gene and muscle fiber size, lean mass, power performance, and power athlete status.

## Materials and methods

### Ethical approval

This study was approved by the Gazi University Non-Interventional Clinical Research Ethics Committee (reference 02; approval date: 18 January 2021) and the Ethics Committee of the Federal Research and Clinical Center of Physical–Chemical Medicine of the Federal Medical and Biological Agency of Russia (reference 2017/04; approval date: 4 July 2017). Written informed consent was obtained from each participant before the start of this study, which complied with the Declaration of Helsinki and ethical standards for sport and exercise science research.

### Participants

#### The Turkish cohort

The present study involved 64 high-level track-and-field athletes, comprising 33 women and 31 men, aged between 18 and 32 years. They voluntarily participated in the study. These athletes, affiliated with various clubs under the Turkish Track and Field Federation, were licensed in different track-and-field disciplines and adhered to rigorous training regimens, practicing at least 6 days a week. The detailed information is provided in Supplementary Table 1. The athletes were categorized into four primary groups based on the characteristics of their events: sprints/throws/jumps (power group; *n* = 17), middle-distance running (*n* = 15), long-distance running (*n* = 13), and racewalking (*n* = 19). This classification was determined by factors such as event distance, duration, and energy expenditure type. All participants held a national ranking within the top ten in their respective disciplines and competed in prestigious international tournaments, including the European Championships, Mediterranean Games, and Balkan Championships. The sprints/throws/jumps category included athletes specializing in events primarily dependent on anaerobic energy systems, such as sprinting and power-focused sports. All Turkish participants were of Caucasian origin.

#### The Russian cohorts

A case–control study included 59 weightlifters (36 males, mean age 26.7 (5.7) years; 23 females, mean age 26.7 (3.0) years) and 162 sprinters (95 males, mean age 26.1 (5.3) years; 67 females, mean age 25.7 (4.5) years). Among the 221 power athletes (weightlifters and sprinters), 50 were world-class athletes (Olympic, World, or European Championship medalists: 16 weightlifters and 34 sprinters), while 171 were elite athletes (international level, non-medalists). None of the athletes had tested positive for doping. The Russian control group consisted of 5451 individuals, as previously described (Barbitoff et al. 2024). All Russian athletes and controls were of Caucasian origin. The muscle fiber size study involved 112 physically active individuals: 35 power-trained males, 45 endurance-trained males, 15 power-trained females, and 17 endurance-trained females. The detailed information is provided in Supplementary Table 2.

#### The FUSION cohort

The gene-expression study (vastus lateralis) included 291 sedentary individuals of European (Finland) descent from the FUSION study, comprising 166 men (mean age 59.5 (8.1) years; height 176.7 (6.7) cm; body mass 87.3 (15.1) kg) and 125 women (mean age 60.3 (8.1) years; height 162.8 (5.6) cm; body mass 71.8 (9.8) kg), as previously described (Taylor et al. 2019).

#### The UK Biobank cohort

The UK Biobank is an open-access, large prospective study that includes phenotypic and genotypic data from more than 500,000 participants (> 90% of whom are of white ethnicity) aged 40–69 years at recruitment (2006–2010) (Sudlow et al. 2015). Of these, 450,243 participants were selected for analysis (appendicular lean mass measured by bioimpedance) (Pei et al. 2020).

### Performance analysis

The International Association of Athletics Federations (IAAF; now World Athletics) scoring system was used to determine the performance levels of the athletes based on their personal best (PB) scores. This system enables the comparison of performances across various track-and-field events and between genders. It provides a standardized method to evaluate and compare the achievements of athletes participating in different track-and-field disciplines (Spiriev 2014).

### BDNF rs6265 genotyping

In the study of Turkish athletes, the oral swabs were collected for *BDNF* gene polymorphism genotyping. Sampling was conducted in collaboration with the hospital where participants underwent routine medical evaluations during the preparatory season. The mouth swab samples were sent to the Gazi University Medical Genetics Laboratory for analysis. The genomic DNA was extracted from oral epithelial cells using the Invitrogen DNA Isolation Kit (Invitrogen, USA). An average of 20 ng of DNA was isolated from each sample and the purity of the isolates was assessed using the OD 260/280 and 260/230 spectrophotometric ratios. Genotyping of the *BDNF* rs6265 polymorphism was performed on all samples using the real-time PCR method (QuantStudio 3; Thermo Fisher Scientific, USA) and TaqMan Genotyping Kits (Catalog No. 4362691; Thermo Fisher Scientific, USA). The reaction mix contained 5 µL of Genotyping Master Mix (Applied Biosystems, Foster City, CA), 5 µL of genotyping mix (Applied Biosystems, USA), 3.5 µL of nuclease-free water (Thermo Fisher, USA), and 1 µL of isolated DNA. Real-time PCR conditions were completed following the supplier’s protocol.

For the Russian athletes, molecular genetic analysis was conducted using DNA extracted from leukocytes obtained from 4 mL of venous blood. DNA extraction and purification were performed using a commercial kit, following the manufacturer’s instructions (Technoclon, Moscow, Russia). Genotyping of the rs6265 polymorphism was carried out using microarray technology (Illumina, San Diego, CA, USA) with HumanOmni1-Quad and HumanOmniExpress BeadChips (Illumina), as described previously (Semenova et al. 2020). Russian control samples were genotyped through whole-exome sequencing, as detailed previously (Barbitoff et al. 2024).

### Gene-expression analysis

The transcriptome analysis of muscle samples from the FUSION cohort was performed using RNAseq, as described previously (Taylor et al. 2019). The expression levels of the *BDNF* and *MTOR* genes are reported in transcripts per kilobase million (TPM).

### Assessment of the cross-sectional area (CSA) of fast-twitch muscle fibers

Muscle fiber composition and the cross-sectional area (CSA) of fast-twitch fibers were evaluated using immunohistochemistry, as previously described (Ahmetov et al. 2009). Briefly, vastus lateralis samples were obtained from the left leg using a modified Bergström needle under local anesthesia (2% lidocaine). Frozen Sects. (7 μm) were prepared and incubated with primary antibodies against slow or fast myosin heavy chain isoforms (M8421, 1:5000; M4276, 1:600; Sigma-Aldrich) for 1 h, followed by secondary FITC-conjugated antibodies (F0257, 1:100; Sigma-Aldrich). After washing and mounting, the images were captured with a fluorescent microscope (Eclipse Ti-U, Nikon). The ratio of stained fibers to the total fiber count was calculated.

### Assessment of physical activity, training parameters, and nutritional status.

Physical activity, training parameters, and nutritional status were assessed using a questionnaire administered to participants in the muscle fiber size study. The questionnaire included information on training frequency, training type (endurance or power), frequency of meat, dairy, and protein intake (per week), and meal frequency (per day). Based on training frequency, athletes were classified into four categories: mildly active (two training sessions per week), moderately active (3–4 sessions per week), highly active (5–7 sessions per week), and extremely active (two sessions per day).

### Statistical analyses

The required sample size for the present study was calculated using G*Power software version 3.1.9.7 (University of Düsseldorf, Germany). A priori power analysis for a *t*-test, appropriate for comparing means across two groups, was conducted. Parameters included an error probability (α) of 0.05, a minimum effect size of 0.7, a comparison ratio of 0.5, and a non-sphericity correction of 1. To achieve a statistical power (1 − β) of 0.80, the analysis determined a minimum sample size of 62 participants, resulting in an actual power of 85.0%. Data were processed and analyzed using the Statistical Package for the Social Sciences (SPSS) software for Mac, version 29.0. The chi-square test assessed Hardy–Weinberg equilibrium (*p* > 0.05) and differences in categorical variables. Data distribution was normal, as indicated by skewness and kurtosis values of − 0.823 and 1.268, respectively. An independent *t*-test was used to analyze differences between two genotypes. The relationships between expression of genes were assessed using regression analysis adjusted for covariates. The scatter plots were generated based on Pearson correlation coefficients. The cross-sectional area of fast-twitch muscle fibers was compared among athletes with different *BDNF* rs6265 genotypes using z-normalization. This method standardizes the data by converting raw values into z-scores, calculated as the difference between an individual value and the group mean, divided by the standard deviation (Field 2013). Z-normalization was applied to account for variability in CSA measurements and ensure comparability across groups. All data are presented as mean (SD), with *p* values < 0.05 considered statistically significant.

## Results

**Fig. 1:**
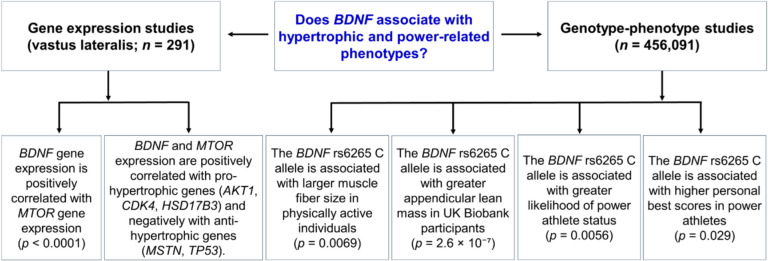
A schematic overview of the study design, cohorts and main findings

A flow diagram illustrating the study design, cohorts, and main findings is shown in Fig. 1.

### Relationship between the expression of the BDNF and MTOR genes

**Fig. 2:**
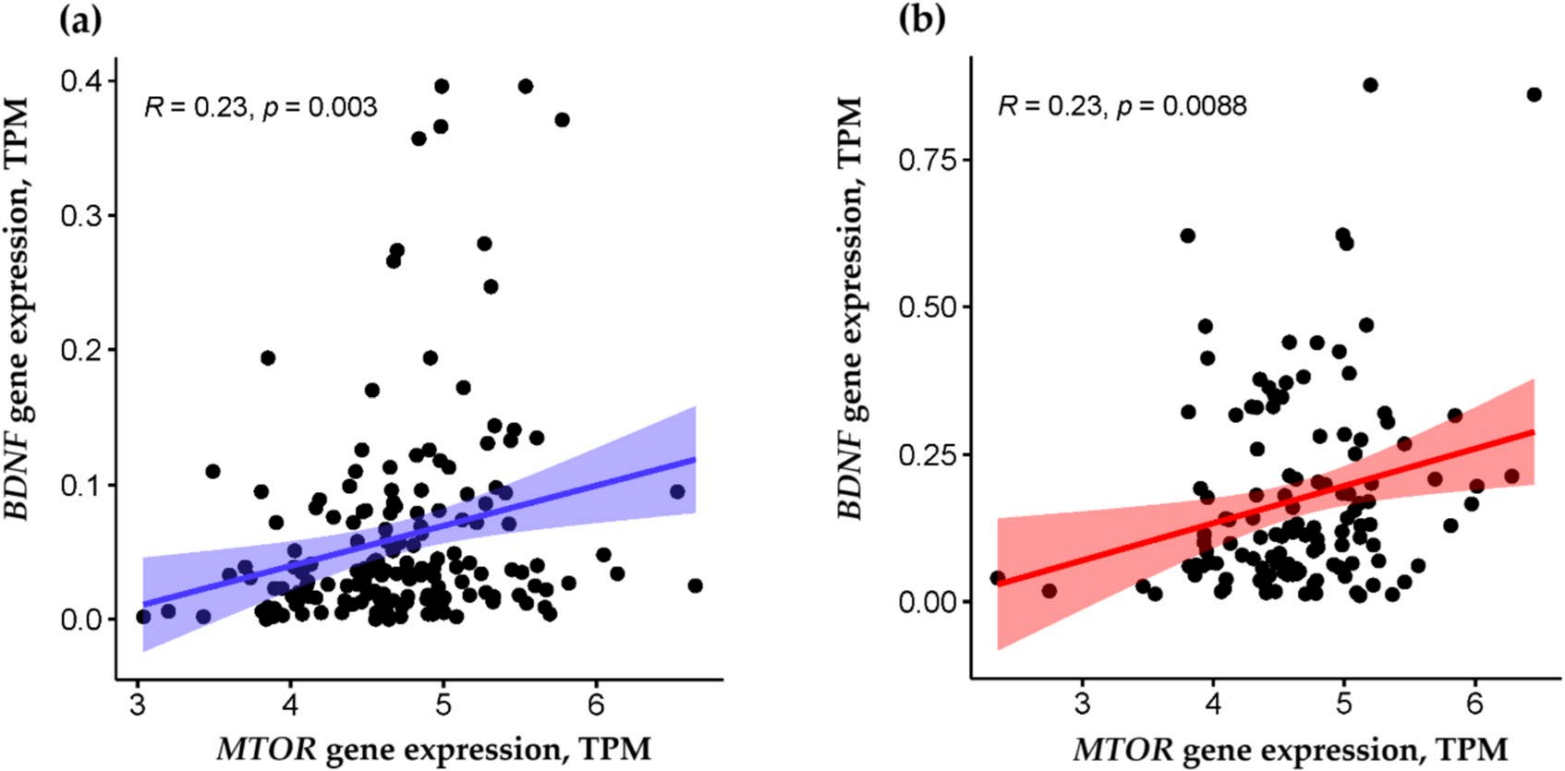
Relationship between the expression of the *BDNF* and *MTOR* genes in (**a**) males and (**b**) females in the FUSION cohort (*n* = 291). *TPM* transcripts per kilobase million, *BDNF* brain-derived neurotrophic factor, *MTOR* mechanistic target of rapamycin kinase

In the FUSION cohort (*n* = 291), females exhibited significantly higher *BDNF* expression than males (0.19 (0.23) vs 0.06 (0.08) TPM, *p* < 0.0001). However, no significant sex differences were observed in *MTOR* gene expression. *BDNF* gene expression was positively associated with *MTOR* gene expression in the m. vastus lateralis in the entire cohort (*p* < 0.0001, adjusted for age, sex, BMI, and smoking status). This positive association remained significant when analyzed separately for males (*r* = 0.23, *p* = 0.003) and females (*r* = 0.23, *p* = 0.0088) (Fig. 2).

Subsequent analyses explored associations between *BDNF* and *MTOR* expression and other genes potentially involved in hypertrophic and mitochondrial pathways (Supplementary Table 3). A positive association was observed between the expression of BDNF and MTOR and pro-hypertrophic genes, including *AKT1*, *CDK4*, and *HSD17B3*, while a negative association was identified with anti-hypertrophic genes, such as *MSTN* and *TP53*. Furthermore, BDNF expression showed a positive relationship with genes involved in mitochondrial biogenesis, including *ESRRA*, *ESRRG*, *GABPB1*, *KDR*, *PPARA*, *PPARGC1A*, *SIRT1*, *TFAM*, *TFB2M*, *TWNK*, and *VEGFA*. A similar pattern of positive associations was observed between MTOR and this same set of mitochondrial biogenesis-related genes (Supplementary Table 3).

### Relationship between genetically predicted BDNF gene expression and athletic performance

There were no statistically significant differences in personal best scores or *BDNF* rs6265 genotype distribution between males and females (data not shown) across sporting disciplines in the Turkish cohort (*n* = 64). Therefore, we justified performing the analysis irrespective of the athletes’ sex. We found that power athletes (i.e., sprinters, throwers, and jumpers) with the *BDNF* CC genotype (predicted to have high *BDNF* expression) had significantly higher personal best scores (*p* = 0.029) compared to carriers of the *BDNF* CT genotype (predicted to have low *BDNF* expression) (Table 1). There were no statistically significant differences in the other groups of track-and-field athletes.

**Table 1:** Differences in personal best scores among Turkish athletes with different *BDNF* rs6265 genotypes across various track-and-field disciplines

Disciplines	*n*	*BDNF* CC (high expression)		*BDNF* CT (low expression)		*P*
		*n*	PB	*n*	PB	
Sprint/throw/jump	17	10	1045 (70)	7	983 (145)	0.029*
Middle distance	15	13	923 (115)	2	834 (220)	0.218
Long distance	13	10	1072 (29)	3	1015 (75)	0.110
Race walk	19	13	1015 (94)	6	895 (106)	0.987

*p* < 0.05 (unpaired *t* test), *PB* personal best score; data are Mean (SD), *BDNF* brain-derived neurotrophic factor

### Differences in BDNF rs6265 allelic frequencies between Russian athletes and controls

All genotype distributions among Russian athletes conformed to Hardy–Weinberg equilibrium (*p* > 0.05). The *BDNF* rs6265 C allele frequency, associated with higher *BDNF* expression, was significantly greater in the overall cohort of power athletes (*p* = 0.0056) compared to controls. This association was also observed individually in weightlifters (*p* = 0.036) and sprinters (*p* = 0.046) (Table 2). However, after adjusting for multiple comparisons, the difference remained statistically significant only in the entire cohort of power athletes (*p*_*adj*_ = 0.017).

**Table 2:** Distribution of *BDNF* rs6265 genotypes and C allele frequencies in Russian athletes and controls

Group	*n*	*BDNF* rs6265 genotypes			C allele frequency, %	*P*
		CC	CT	TT		
Weightlifters	59	49	10	0	91.5	0.036*
Sprinters	162	128	31	3	88.0	0.046*
All power athletes	221	177	41	3	89.4	0.0056*
Russian controls^§^	5,451	NA	NA	NA	84.5	-

*p* < 0.05 (χ^2^ test) indicates statistically significant differences in C allele frequency between athletes and controls. *NA* not available. ^§^Data sourced from the RUSeq database (2025)

### Association between the BDNF rs6265 variant and muscle fiber cross-sectional area in athletes and appendicular lean mass in the UK Biobank cohort

In the muscle fiber size study (*n* = 112), male and female power athletes had a significantly greater cross-sectional area (CSA) of fast-twitch muscle fibers than male and female endurance athletes. Furthermore, within the same training category (power or endurance), male athletes had a significantly greater CSA of fast-twitch muscle fibers compared to female athletes (Supplementary Table 2). To further investigate the effect of the *BDNF* rs6265 variant on muscle fiber size, we performed separate analyses for four subgroups of athletes: female endurance athletes, female power athletes, male endurance athletes, and male power athletes. Additionally, we conducted an analysis combining the four subgroups, adjusting for sex and type of training. Fast-twitch muscle fibers were selected for analysis because they are better predictors of muscle strength than slow-twitch muscle fibers (Verdijk et al. 2010).

**Fig. 3:**
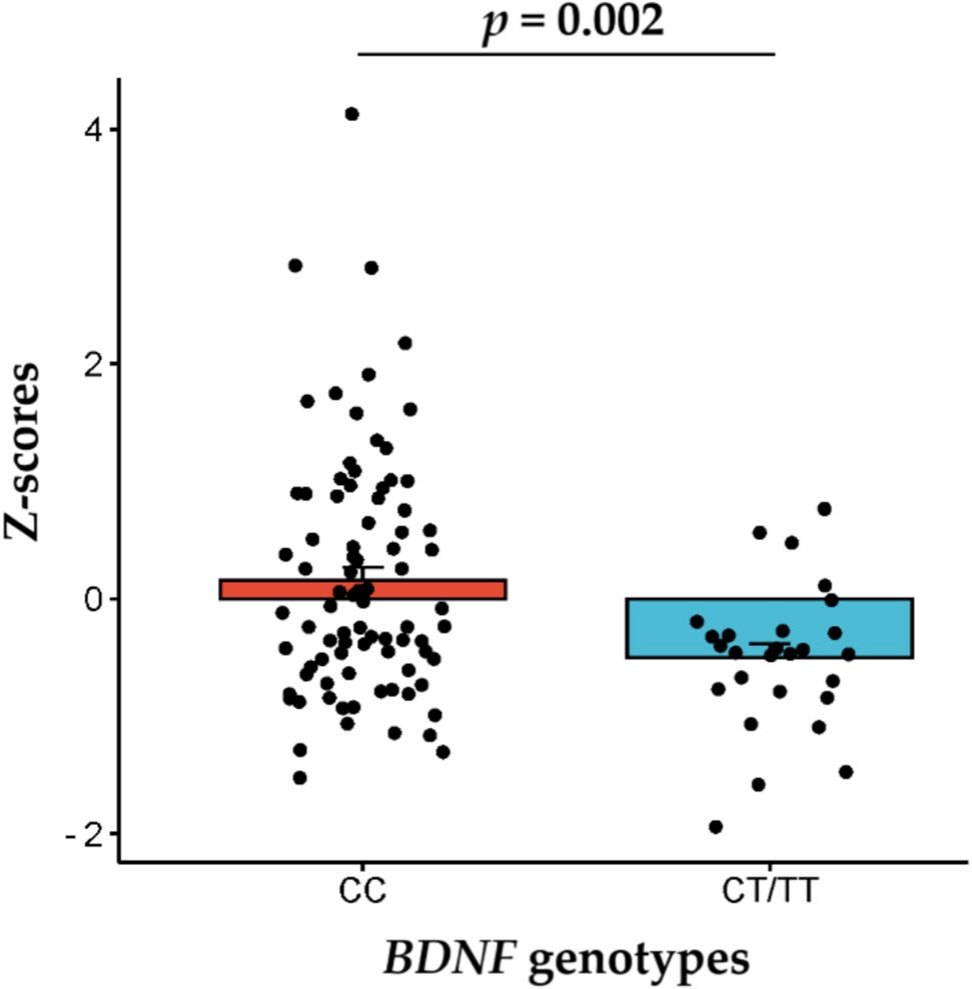
Comparison of the z-normalized cross-sectional area of fast-twitch muscle fibers between athletes with different *BDNF* rs6265 genotypes (*n* = 112)

In the entire cohort, the carriers of the *BDNF* rs6265 CC genotype (predicted to have high *BDNF* expression) exhibited a significantly greater CSA of fast-twitch muscle fibers compared to carriers of the T allele (*p* = 0.002, adjusted for sex and type of training) (Fig. 3). This association remained significant (*p* = 0.0069) after adjusting for additional factors, including age, training frequency, intake of meat, dairy, and protein, as well as meal frequency. On an individual basis, significant differences between genotype groups (CC vs CT + TT) were observed only in male endurance athletes (*p* = 0.039) (Supplementary Table 4).

In silico analysis revealed that the rs6265 C allele was associated with greater appendicular lean mass (a proxy for muscle mass) in the UK Biobank cohort (*n* = 450,243; beta = 0.0124, *p* = 2.6 × 10⁻⁷) (Open targets, 2025).

## Discussion

This study investigated the relationship between *BDNF* and *MTOR* gene expression in human skeletal muscle and examined the association between genetically predicted higher *BDNF* expression and key muscle-related phenotypes, including muscle fiber size, lean mass, power performance, and power athlete status. Our findings provide further evidence supporting the role of *BDNF* in skeletal muscle physiology and athletic performance.

Our study’s key and novel finding was the positive association between genetically predicted higher *BDNF* expression (i.e., carrying the C allele of the rs6265 polymorphism) and skeletal muscle hypertrophy-related phenotypes. Specifically, we observed that athletes with the CC genotype had a greater cross-sectional area of fast-twitch muscle fibers. Furthermore, in silico analysis using data from the UK Biobank revealed a positive association between the C allele and appendicular lean mass, a surrogate for muscle mass.

One potential mechanism that may explain the observed associations is the connection between BDNF and the mTOR signaling pathway, a key regulator of protein synthesis and muscle growth. Giacco et al. (2022) demonstrated that mild endurance exercise in rats activated BDNF-mTOR signaling, enhancing muscle quality through downstream Akt-mTOR pathways. Similarly, Hang et al. (2017) showed that BDNF activated Akt and preserved mTOR phosphorylation in cardiac tissue, highlighting its role in promoting cellular survival and function. Suijo et al. (2013) found that resistance exercise increased BDNF expression and activated the mTOR-p70S6K pathway, correlating with muscle protein synthesis and improved cognitive function. Finally, Chen et al. (2009) revealed that BDNF-induced activation of the PI3K-Akt-mTOR pathway modulated synaptic plasticity, suggesting broader physiological implications.

In addition to these observations, our study also demonstrated a significant association between *BDNF* and *MTOR* gene expression in the m. vastus lateralis, detected in both males and females. This finding suggests that BDNF may influence muscle growth and adaptation, at least in part, by modulating *MTOR* expression. While the exact mechanisms of this interaction require further investigation, several possibilities exist. BDNF signaling through its TrkB receptor can activate downstream signaling cascades, including the PI3K/Akt pathway, which in turn can stimulate mTOR complex 1 (mTORC1) activity (Schiaffino et al. 2021). Alternatively, BDNF may influence mTOR signaling indirectly through other pathways involved in muscle metabolism and adaptation. Moreover, BDNF has been shown to promote muscle regeneration by enhancing satellite cell proliferation and differentiation (Clow and Jasmin 2010). This connection could lead to increased muscle fiber size and improved muscle repair following exercise.

Using transcriptome data from the FUSION cohort, we also identified a positive association between the expression of *BDNF* and *MTOR* and pro-hypertrophic genes (*AKT1, CDK4,* and *HSD17B3*), along with a negative association with anti-hypertrophic genes (*MSTN* and *TP53*), which supports our hypothesis that the *BDNF* exerts a hypertrophic effect on human skeletal muscles. Additionally, we observed a positive relationship between *BDNF* and genes involved in mitochondrial biogenesis, including *ESRRA, ESRRG, GABPB1, KDR, PPARA, PPARGC1A, SIRT1, TFAM, TFB2M, TWNK,* and *VEGFA*. A similar positive association was found between *MTOR* and the same set of genes. These findings align with existing literature, where *BDNF* has been shown to promote mitochondrial biogenesis through the activation of the PI3K/Akt and MAPK/ERK signaling pathways, leading to the induction of *PPARGC1A*, which subsequently upregulates mitochondrial genes such as *TFAM, ESRRA,* and *GABPB1*, essential for mitochondrial DNA transcription and replication (Marosi and Mattson 2014). Similarly, *MTOR* regulates mitochondrial biogenesis through the activation of mTORC1, which enhances the transcription of *PPARGC1A* and its downstream targets, including *TFAM, SIRT1,* and *VEGFA*, facilitating mitochondrial DNA replication, and improving oxidative capacity (Cunningham et al. 2007; Morita et al. 2015). Overall, our transcriptome analysis highlights a strong concordance between the biochemical pathways regulated by *BDNF* and *MTOR*.

Our findings also indicate that genetically predicted higher *BDNF* expression is positively associated with personal best scores in power events and power athlete status. These results are consistent with previous reports linking the *BDNF* rs6265 polymorphism to power and sprint performance. In a study of 535 elite male youth soccer players, *BDNF* rs6265 CC homozygotes were found to sprint faster and jump further compared to T allele carriers, suggesting a potential genetic influence on speed and power performance (Murtagh et al. 2020). While the *BDNF* rs6265 (Val66Met) polymorphism has been extensively studied concerning cognitive traits (Miyajima et al. 2008; Kambeitz et al. 2012; Kennedy et al. 2015; Boots et al. 2017), personality (Humińska-Lisowska et al. 2024), and physical activity (Ahmetov et al. 2024), its emerging role in influencing athletic performance and serving as a biomarker in specific sports disciplines provides a novel perspective on its broader biological significance (Bulğay et al. 2020).

The association between the *BDNF* rs6265 polymorphism, power performance, and athletic status may be partially attributed to the polymorphism’s effect on muscle fiber size and lean mass, as muscle size is directly linked to strength, sprinting, and jumping performance (Seynnes et al. 2007; Handsfield et al. 2017; Bchini et al. 2023). Furthermore, BDNF’s role in neuromuscular function, including its ability to enhance synaptic transmission and promote long-term potentiation (De Vincenti et al. 2019; Miranda et al. 2019; Andreska et al. 2020), may further contribute to improved power output and athletic performance. The animal studies have also reported a shift toward a fast-twitch muscle fiber phenotype with higher *Bdnf* expression, which may underlie improvements in power-related traits (Delezie et al. 2019). Female mice with muscle-specific *Bdnf* deficiency also exhibited impaired fuel switching to fatty acids during fasting, reducing ATP production, myofiber necrosis, diminished muscle strength, and insulin resistance (Yang et al. 2019). These findings highlight the critical role of BDNF in maintaining muscle function and metabolic flexibility.

A major strength of this study is the integration of genetic, transcriptomic, and phenotypic data across diverse cohorts, which provides robust evidence for the role of BDNF in skeletal muscle traits. Additionally, the large sample size in the UK Biobank cohort strengthens the validity of our findings, decreasing the chances of obtaining false-positive results. Including both athlete and general population samples enhances the generalizability of our findings. However, several limitations should be acknowledged. First, while our study identified associations, causal relationships cannot be definitively established. Further research is needed to fully elucidate the molecular pathways through which BDNF influences muscle physiology and athletic performance. Future studies should also investigate the interplay between BDNF, mTOR, and other relevant signaling pathways in skeletal muscle using in vitro and in vivo models. Longitudinal studies examining the effects of exercise interventions on *BDNF* expression and muscle adaptations in individuals with different *BDNF* genotypes are also warranted. Second, we acknowledge that mRNA and protein levels do not always align due to post-transcriptional and post-translational regulation, and assessing protein content could indeed enhance our findings. However, according to existing literature, directly measuring BDNF protein levels in human skeletal muscle is challenging due to its low abundance (Edman et al. 2024). In fact, based on our unpublished observations, BDNF protein expression was detectable in the vastus lateralis muscle in only 30% of participants. Therefore, we focused on transcript-level analysis, where we observed a robust positive correlation between *BDNF* and *MTOR* gene expression. Third, the genetic analyses were limited to the *BDNF* rs6265 variant, and other genetic factors influencing *BDNF* expression and function were not explored. Fourth, the sample size for some of the athlete-specific analyses, particularly the personal best scores, was relatively small, which may limit the statistical power. Robust findings in the field of exercise genomics can only be achieved through extensive collaboration and data sharing among researchers (Eynon et al. 2013). Finally, while the UK Biobank analysis provided robust statistical power, it relied on appendicular lean mass as a proxy for muscle mass, which may not perfectly reflect skeletal muscle size.

## Conclusions

In conclusion, our findings provide further evidence for the role of BDNF in skeletal muscle physiology and athletic performance. We have demonstrated a positive correlation between *BDNF* and *MTOR* gene expression in human skeletal muscle, with genetically predicted higher *BDNF* expression associated with greater muscle fiber size, enhanced power performance, and power athlete status. These findings underscore the significance of the BDNF-mTOR axis in muscle biology, highlighting BDNF as one of the key regulators of muscle mass and physical performance. They suggest that genetic variation in the *BDNF* gene may contribute to individual differences in muscle fiber size, related traits, and athletic potential. This growing body of evidence provides a foundation for future research aimed at optimizing muscle function and athletic performance through targeted interventions.

## Supplementary Information

Below is the link to the electronic supplementary material.

Supplementary file1 (DOCX 26 KB)

## Data Availability

The data presented in this study are available on request from the corresponding author.

## Abbreviations

AKT1: AKT serine/threonine kinase 1
BDNF: Brain-derived neurotrophic factor
CDK4: Cyclin dependent kinase 4
CSA: Cross-sectional area of muscle fibers
DNA: Deoxyribonucleic acid
ESRRA: Estrogen related receptor alpha
ESRRG: Estrogen related receptor gamma
GABPB1: GA binding protein transcription factor subunit beta 1
HSD17B3: Hydroxy steroid 17-beta dehydrogenase 3
IAAF: International Association of Athletics Federations
KDR: Kinase insert domain receptor
MSTN: Myostatin
mTOR: Mechanistic target of rapamycin kinase
mTORC1: MTOR complex 1
PPARA: Peroxisome proliferator activated receptor alpha
PPARGC1A: PPARG coactivator 1 alpha
SIRT1: Sirtuin 1
SNP: Single nucleotide polymorphism
TFAM: Transcription factor A, mitochondrial
TFB2M: Transcription factor B2, Mitochondrial
TP53: Tumor protein P53
TPM: Transcripts per kilobase million
TWNK: Twinkle MtDNA helicase
VEGFA: Vascular endothelial growth factor A
