# Supplementary material for: BDNF coexpresses with MTOR and is associated with muscle fiber size, lean mass and power-related traits
Source: Eur J Appl Physiol. 2025 Apr 29;125(10):2781–92. doi: 10.1007/s00421-025-05804-3 (PMC12479623; doi:10.1007/s00421-025-05804-3)
Supplement: Supplementary file 1 — Supplementary file1 (DOCX 26 KB) [file 421_2025_5804_MOESM1_ESM.docx]

***BDNF* coexpresses with *MTOR* and is associated with muscle fiber size, lean mass and power-related traits**

**Supplementary file**

**Supplementary Table 1.** Characteristics of Turkish track-and-field athletes

| Variables | Disciplines | n | X̄ | SD |
| --- | --- | --- | --- | --- |
| Age (years) | Sprint/throw/jump | 17 | 23.35 | 4.13 |
|  | Middle-distance | 15 | 20.86 | 3.73 |
|  | Long-distance | 13 | 25.30 | 5.89 |
|  | Racewalk | 19 | 19.68 | 2.88 |
| Height (cm) | Sprint/throw/jump | 17 | 176.23 | 6.43 |
|  | Middle-distance | 15 | 173.20 | 7.18 |
|  | Long-distance | 13 | 169.61 | 10.82 |
|  | Racewalk | 19 | 167.57 | 10.62 |
| Body weight (kg) | Sprint/throw/jump | 17 | 70.00 | 12.50 |
|  | Middle-distance | 15 | 59.64 | 8.25 |
|  | Long-distance | 13 | 7.80 | 3.70 |
|  | Racewalk | 19 | 55.15 | 11.87 |
| Sport experience (year) | Sprint/throw/jump | 17 | 11.76 | 4.5 |
|  | Middle-distance | 15 | 7.80 | 3.70 |
|  | Long-distance | 13 | 11.46 | 4.66 |
|  | Racewalk | 19 | 7.89 | 3.08 |
| PB (sn or cm) | Sprint/throw/jump | 17 | 1019.52 | 107.57 |
|  | Middle-distance | 15 | 910.73 | 125.94 |
|  | Long-distance | 13 | 1027.76 | 70.70 |
|  | Racewalk | 19 | 997.10 | 110.78 |

X̄, Mean; SD, Standard Deviation; PB, personal best scores.

**Supplementary Table 2.** Cross-sectional area of muscle fibers in the m. vastus lateralis of athletes.

| Group | *n* | Age, years | Height, cm | Weight, kg | CSA of fast-twitch muscle fibers, µm^2^ | *P* value for the differences between P and E | *P* value for the differences between M and F | CSA of slow-twitch muscle, µm^2^ fibers | *P* value for the differences between P and E | *P* value for the differences between M and F |
| --- | --- | --- | --- | --- | --- | --- | --- | --- | --- | --- |
| Male power | 35 | 27.2 (6.4) | 180.0 (6.8) | 87.9 (11.1) | 7696 (2252) | 0.0002* | <0.0001* | 5850 (1142) | 0.334 | 0.0096* |
| Male endurance | 45 | 34.5 (8.5) | 180.4 (6.1) | 77.7 (8.9) | 5869 (1886) |  | 0.0004* | 5553 (1496) |  | 0.016* |
|  |  |  |  |  |  |  |  |  |  |  |
| Female power | 15 | 25.6 (5.8) | 169.3 (4.6) | 61.8 (6.0) | 4947 (1043) | 0.034* | - | 4908 (1100) | 0.369 | - |
| Female endurance | 17 | 29.2 (8.6) | 168.0 (7.1) | 57.8 (5.1) | 3960 (1416) |  | - | 4587 (897) |  | - |

**p* < 0.05, statistically significant differences (unpaired *t* test). Data are mean (SD); P, power athletes; E, endurance athletes; M, male athletes, F, female athletes; CSA, cross-sectional area.

**Supplementary Table 3.** Relationship between *BDNF* and *MTOR* expression and other genes potentially involved in hypertrophic and mitochondrial pathways.

| **Genes** | **Relationship with *BDNF* gene expression** | | **Relationship with *MTOR* gene expression** | |
| --- | --- | --- | --- | --- |
|  | P value | Coefficient | P value | Coefficient |
| **Pro-hypertrophic genes** | | | | |
| *AKT1* | 0.0135* (males) | 0.017 | 0.0348* | 0.084 |
| *HSD17B3* | 0.0013* | 0.2062 | 0.143 | 0.467 |
| *CDK4* | <0.0001* | 0.028 | 0.0095* | 0.075 |
| **Anti-hypertrophic genes** | | | | |
| *MSTN* | 0.0019* (males) | -0.007 | 0.022* | -0.035 |
| *TP53* | 0.263 | -0.018 | 0.021* | -0.184 |
| **Genes involved in mitochondrial biogenesis** | | | | |
| *ESRRA* | 0.016* | 0.002 | <0.0001* | 0.023 |
| *ESRRG* | <0.0001* | 0.052 | <0.0001* | 0.304 |
| *GABPB1* | 0.028* | 0.043 | 0.0184* | 0.229 |
| *KDR* | 0.0003* | 0.03 | <0.0001* | 0.177 |
| *PPARA* | <0.0001* | 0.033 | <0.0001* | 0.226 |
| *PPARGC1A* | <0.0001* | 0.016 | <0.0001* | 0.112 |
| *SIRT1* | 0.0077* | 0.014 | 0.0027* | 0.08 |
| *TFAM* | 0.0015* | 0.02 | <0.0001* | 0.131 |
| *TFB2M* | 0.001* | 0.013 | <0.0001* | 0.078 |
| *TWNK* | <0.0001* | 0.079 | <0.0001* | 0.795 |
| *VEGFA* | <0.0001* | 0.0068 | <0.0001* | 0.051 |

**p* < 0.05, statistically significant relationships between gene expression levels after adjusting for covariates.

**Supplementary Table 4.** Differences in the cross-sectional area of fast-twitch muscle fibers in the m. vastus lateralis among athletes of different *BDNF* genotype groups.

| Group | *BDNF* rs6265 genotypes | | | | *P* value |
| --- | --- | --- | --- | --- | --- |
|  | CC | | CT+TT | |  |
|  | *n* | CSA, µm^2^ | *n* | CSA, µm^2^ |  |
| Male power | 27 | 7908 (2415) | 8 | 6982 (1489) | 0.314 |
| Male endurance | 34 | 6197 (1932) | 11 | 4856 (1359) | 0.039* |
|  |  |  |  |  |  |
| Female power | 11 | 5244 (1031) | 4 | 4131 (566) | 0.065 |
| Female endurance | 13 | 4173 (1565) | 4 | 3268 (228) | 0.277 |

**p* < 0.05, statistically significant differences (unpaired *t* test). Data are mean (SD). CSA, cross-sectional area.
